# Occurrence of Toxic Metals and Metalloids in Muscle and Liver of Italian Heavy Pigs and Potential Health Risk Associated with Dietary Exposure

**DOI:** 10.3390/foods11162530

**Published:** 2022-08-21

**Authors:** Sergio Ghidini, Maria Olga Varrà, Lenka Husáková, Giovanni Loris Alborali, Jan Patočka, Adriana Ianieri, Emanuela Zanardi

**Affiliations:** 1 Department of Food and Drug, University of Parma, Strada del Taglio 10, 43126 Parma, Italy; 2 Department of Analytical Chemistry, Faculty of Chemical Technology, University of Pardubice, Studentska 573 HB/D, CZ-532 10 Pardubice, Czech Republic; 3 Istituto Zooprofilattico Sperimentale della Lombardia e dell’Emilia-Romagna, Via A. Bianchi 9, 25124 Brescia, Italy

**Keywords:** meat inspection, toxic metals, food safety, toxicity, exposure assessment, chemical risk

## Abstract

Muscle and liver tissues from Italian heavy pigs were analyzed to investigate whether the chronic consumption of these products by local consumers could represent a health risk in relation to the contamination by some toxic metals and metalloids (TMMs). The concentrations of Al, As, Cd, Cr, Cu, Fe, Ni, Pb, Sn, U, and Zn were measured with an inductively coupled plasma–mass spectrometer, while Hg analysis was performed by using a mercury analyzer. Fe, Zn, and Cu were the most abundant elements in both tissues, while U was detected only at ultra-trace levels. As, Cd, Cu, Fe, Hg, Pb, U, and Zn showed significantly higher concentrations in livers compared to muscles (*p* ≤ 0.01), with Cd and Cu being 60- and 9-fold more concentrated in the hepatic tissue. Despite this, concentrations of all TMMs were found to be very low in all the samples to the point that the resulting estimated dietary intakes did not suggest any food safety concern. Indeed, intakes were all below the toxicological health-based guidance values or resulted in low margins of exposure. Nevertheless, in the calculation of the worst-case exposure scenario, the children’s estimated intake of Cd, Fe, and Zn through the sole consumption of pig liver contributed to more than 23, 38, and 39% of the tolerable weekly intakes of these elements, while the combined consumption of pig liver and pig muscle to more than 24, 46, and 76%. These findings alert about the probability of exceeding the toxicological guidance values of Cd, Fe, and Zn though the whole diet, suggesting long-term negative health effects for the younger population.

## 1. Introduction

Many chemical contaminants widely distributed in the environment, including toxic metals and metalloids (TMMs), can enter the food chain, persist, and bioaccumulate to critical concentrations that may pose a long-term risk to human health. Sources of contamination of food by TMMs are many and varied, but most result from anthropogenic emissions such as those related to mining activities, smelters, fossil fuel combustion, pesticide use, combustion of by products, and traffic [1,2]. Among TMMs, non-essential trace elements such as As, Cd, Hg, and Pb, but also essential trace elements at high concentrations such as Cr, Cu, Fe, and Zn, are of primary concern due to their toxicological impact in both humans and animals. Long-term exposure to TMMs can lead to a wide spectrum of potential adverse health effects, such as kidney and liver diseases, central and peripheral nervous system and circulatory dysfunctions, reproductive toxicity, genotoxicity, teratogenicity, and carcinogenicity [3]. Due to their occurrence, toxicity, and potential for human exposure, As, Pb, Hg, and Cd still rank 1st, 2nd, 3rd, and 7th, respectively, in the list of prioritized contaminants drawn up by the Agency for Toxic Substances and Disease Registry and Environmental Protection Agency in the USA [4].

TMMs are carried over to high concentration levels from soil, air, feed, and water to animals and then to derived animal-based foods such as fishery products, meat, and meat products [3]. The high levels of contamination, together with the high rate of dietary consumption of these products, are therefore responsible for human exposure to TMMs at levels which can lead to adverse health outcomes [5,6,7]. In this context, concentration levels of TMMs in tissues of wild animals are mainly related to environmental pollution, while contamination levels in tissues of farmed animals (especially those from intensive livestock facilities) are ascribed to feed, in which additives and mineral supplements containing high concentrations of toxic metals can be present [8,9,10,11].

Among farmed livestock meat, pig meat (as fresh or industrial processed products) is very popular and highly consumed in Europe [12]. The amount of undesirable toxic elements in pig skeletal muscle tissues is not generally a source of concern for food safety. However, the same is not always true for edible internal organs, since most TMMs have a particular tropism for kidney, liver, heart, lungs, and brain where they undergo progressive accumulation [13,14]. This aspect may be relevant from the perspective of human health, since in many countries of the world, including Europe, pig offal and, in particular, liver, is widely appreciated and largely consumed as part of the standard diet.

Data on TMM concentrations in pig meat and liver were reported from different countries. Mostly, contamination levels were measured in samples of lean pigs slaughtered at 5–6 months of age and weighing around 100 kg. These samples were generally found to be within the recommended safety limits [13,15,16,17,18,19,20], with fewer studies advising about a potential health risk for consumers [5,14,21,22]. On the contrary, there is a huge lack of information about TMM contamination in the Italian heavy pig production chain, which, standing out for the fattening of pigs up to 170 kg and their slaughter at 9 months of age, would call for research investigating whether the longer lifespan and the higher slaughter weights are associated with higher TMM concentrations in tissues and with the potential accumulation to harmful levels for human health.

Therefore, in the present study, the degree of contamination by 12 TMMs (As, Al, Cd, Cr, Cu, Fe, Hg, Ni, Pb, Sn, U, and Zn) of pig muscle and liver tissues obtained from the Italian heavy pig production chain was evaluated for the first time and employed to perform a dietary exposure assessment of different age groups of the Italian population. The impact of pig meat and pig liver consumption on the toxicological guidance values of each TMM was also estimated to characterize the potential human health risk associated with the dietary exposure to these food contaminants.

## 2. Materials and Methods

### 2.1. Sampling and Sample Preparation

The sampling of muscle and liver tissues from fattening heavy pigs (aged 9 months and weighing approximately 160 kg live weight) was performed directly from a slaughter line in a major abattoir of northern Italy. Specifically, crossbred heavy pigs in line with the Parma Ham Protected Designation of Origin (PDO) consortia requirements were all from intensive indoor farms of northern Italy. To ensure representativeness of the sample population, a random sampling was carried out: 16 different pig farms located in the Parma Ham PDO production area were considered and 5 pigs were selected from each farm (for a total of 80 animals).

The diaphragm muscle (70~100 g) and the right lateral lobe of the liver (400~500 g) were excised from each animal. While livers were chosen due to representing the most frequently consumed pig offal in Italy, the decision to collect diaphragm samples was driven by economic and technical reasons and based on previous agreement with the abattoir. Indeed, meat from diaphragm is considered less competitive compared to other skeletal meat cuts from Italian heavy pig carcasses, which are mainly destined for the production of high-quality PDO meat products.

Visible connective and fat tissues were discarded from the muscle samples, while a sub-portion of the liver was excised by cutting through the middle of the liver lobe. Each sample was minced by a ceramic knife and sub-samples of approx. 30 g were stored at −80 °C for at least 24 h before being freeze-dried at −55 °C for 24 h by means of a LyoQuest −55 Plus freeze-dryer (Telstar Co., Terrassa, Barcelona, Spain). After freeze-drying, the samples were powdered and analyzed for TMMs.

### 2.2. Moisture Analysis

Moisture content of muscle and liver samples was measured in fresh minced aliquots according to the air-drying reference method AOAC 950.46 B. with slight modifications [23]. Briefly, approx. 5 g of samples (annotated weight) were evenly placed on 100 mm diameter ceramic dishes and dried in a forced air oven at 102 °C for about 16 h. Samples were then cooled under a desiccator to constant weight and the moisture content reported as percentage of loss in weight calculated from the fresh wet sample weight and the dried sample weight. Average moisture contents of muscles and livers were 73.1 ± 3.3% and 70.7 ± 2.1%, respectively.

### 2.3. Quantification of TMMs

Total Hg was directly quantified in pulverized samples (50 mg approx.) without prior digestion via single-purpose atomic absorption spectroscopy using an AMA-254 Advanced Mercury Analyzer (Altec Ltd., Prague, Czech Republic) with wavelength reading at 253.6 nm. The analytical parameters and working conditions of the instrument were as follows: 99.5% oxygen flow rate, 170 mL/min; drying temperature/time, 120 °C/60 s; decomposition temperature/time, 750 °C/150 s; Hg release temperature/time, 900 °C/45 s; Hg reading time, 60 s.

The Agilent 7900 inductively coupled plasma-mass spectrometer (ICP-MS, Agilent Technologies, Inc., Santa Clara, CA, USA) fitted with standard nickel cones, glass concentric nebulizer MicroMist (400 µL min^−1^), the Peltier-cooled (2 °C) quartz spray chamber, and 2.5 mm internal diameter quartz torch [24] was used for the quantification of As, Al, Cd, Cr, Cu, Fe, Ni, Pb, Sn, U, and Zn. The instrument was equipped with an octopole-based collision cell for effective and reliable removal of multiple polyatomic interferences using kinetic energy discrimination in a standard helium (“He”) or high-energy helium (“HE He”) mode. Instrument operating parameters are given in Supplementary Table S1.

Prior to ICP-MS analyses, the samples were digested in a Speed wave XPERT closed microwave oven system (Berghof, Eningen, Germany) with the power output of the magnetron of 1450 W and optical sensors for contactless real-time recording of the sample temperature and pressure in each vessel. The high-pressure-resistant (up to 100 bar) TFM™-PTFE DAC100 vessels were used for sample digestion in combination with the Berghof Multitube System to increase the sample throughput [25]. In this case, 100 mg of pulverized sample were fully mineralized after addition of 4 mL of 16% HNO_3_ (Lach-Ner, Neratovice, Czech Republic) and 1 mL of 30% H_2_O_2_ (Fluka Chemie AG, Buchs, Switzerland) at 180 °C for 20 min (5 min ramp), 220 °C for 20 min (5 min ramp), and 100 °C for 5 min (5 min ramp). Following digestions, samples were diluted to 25 mL with 0.05 μS cm^−1^ resistivity ultrapure water (Milli-Q^®^ system, Millipore Corporation, Bedford, MA, USA) and then then subjected to elemental analysis by ICP-MS. Each sample was prepared in three replicates. Blanks, consisting of deionized water, and reagents were subjected to a similar preparation procedure.

Quantification of TMMs by ICP-MS was performed using multi-element calibration curves which were obtained by analyzing calibration solutions at five different concentrations of standards. Standard solutions were obtained by dilution of commercial single- or multi-element standard solutions (Merck, Darmstadt, Germany; Analytika Ltd., Prague, Czech Republic; SCP Science, Montreal, QC, Canada). Linear calibrations with coefficients of determination >0.998 were obtained for all elements. To compensate for possible instrumental drift and matrix effects, a 200 µg L^−1^ internal standard solution of Rh was simultaneously aspired and mixed with samples.

#### Validation of the Analytical Method

The trueness, intra-day, and inter-day assay precisions were evaluated by analyzing different certified reference materials (CRMs) covering all the elements under investigation: NIST SRM 1577 Bovine Liver (National Institute of Science and Technology, NIST, Gaithersburg, MD, USA); BCR^®^ 184 Bovine Muscle (Institute for Reference Materials and Measurements, IRMM, Geel, Belgium); BCR^®^ 185 Bovine Liver (IRMM, Geel, Belgium); NCS ZC73015 Milk Powder (National Research Centre for Certified Reference Materials, NRCRM, Beijing, China); CRM 12-2-04 Essential and Toxic Elements in Wheat Bread Flour (pb-anal, Kosice, Slovakia); CRM 12-2-03 P-Alfalfa Essential and Toxic Elements in Lucerne (pb-anal, Kosice, Slovakia); CRM 12-02-01 Bovine Liver (pb-anal, Kosice, Slovakia). The obtained recovery values ranged from 90 to 111% for all the elements tested, as reported in Table S2 of the Supplementary Materials, documenting excellent accuracy and interference removal. The RSD values of intra-day and inter-day studies were mostly found to be below 10%, thus showing a good precision of the method (Table S2).

The method limits of detection (MLODs) and limits of quantification (MLOQs) were calculated, respectively, as 3 and 10 times the standard deviation of 10 consecutive blank samples divided by the slope of the calibration curve and considering the dilution factors used during the sample preparation steps. The obtained results are reported in Table S3 of the Supplementary Materials.

### 2.4. Data Processing

For descriptive statistics, elemental concentrations referring to freeze-dried samples were transformed to fresh sample concentrations according to the average moisture contents of muscles or livers and expressed as mg kg^−1^ or µg kg^−1^ wet weight (see Section 2.2).

Around 24% and 0.4% of left-censored results (data points below the MLODs) referring to experimental concentration of As were found in muscles and liver samples, respectively. Since missing values were below the threshold of 60%, the substitution method recommended by EFSA and the World Health Organization (WHO) was applied [26,27]. As described below in Section 2.5.2, values below the MLOD were therefore replaced by half of the MLOD values (to further estimate a less conservative human exposure scenario) or MLOD (to further estimate the worst-case exposure scenario), while values below the MLOQ were numerically reported.

Statistical analysis was performed by using OriginPro 2021 software (v. 9.8.0.200, OriginLab Corporation, Northampton, MA, USA). Data were evaluated for normality and homoscedasticity by using the Shapiro–Wilk’s and the Levene’s tests. Since none of the investigated elements was found to follow a normal distribution and the homogeneity of variance was confirmed only for Al, As, Hg, and Ni, the non-parametric Mann–Whitney U test was used to compare median values of these elements between the two pig tissues. A *p*-value lower than 0.01 was selected as the threshold for statistical significance.

### 2.5. Exposure and Health Risk Assessment of TMMs

#### 2.5.1. Retrieval of Toxicological Reference Values and Reference Points

For all the analyzed TMMs, toxic effects have been documented and toxicological reference values (health-based guidance values, HBGVs) or reference points (benchmark dose lower limits, BMDLs) officially established as thresholds of concern by the European Food safety Authority (EFSA) and the Joint FAO/WHO Expert Committee on Food Additives (JECFA). While HBGVs correspond to maximum doses or concentrations of a toxic substance to which individuals can be exposed over a specific period of time without appreciable health risks, BMDLs are defined as points on an experimental dose–response relationship for critical effects and correspond to doses of toxic compounds determining a percentage increase (1, 5, or 10%) of adverse health effects [28]. HBGV and BMDL values were retrieved from the JECFA flavors, food additives, contaminants, toxicants, and veterinary drugs online database [29] and the online EFSA Chemical Hazards Database OpenFoodTox [30]. They included: tolerable daily intake (TDI) for Ni (13 µg kg bw^−1^ day^−1^), Cr(III) (0.3 mg kg bw^−1^ day^−1^), and U (0.6 µg kg bw^−1^ day^−1^); provisional maximum tolerable daily intake (PMTDI) for Cu (0.5 mg kg bw^−1^ day^−1^), Fe (0.8 mg kg bw^−1^ day^−1^), and Zn (0.3^−1^ mg kg bw^−1^ day^−1^) [29]; tolerable weekly intake (TWI) for Al (1 mg kg bw^−1^ week^−1^), Cd (2.5 µg kg bw^−1^ week−1), and inorganic Hg (*i*Hg) (4 µg kg bw^−1^ week); provisional tolerable weekly intake (PTWI) for Sn (14 mg kg bw^−1^ week) [29]; BMDL for inorganic As (*i*As) (BMDL_01_: 0.69, 3.7, and 7.5 µg kg bw^−1^ day^−1^ for 1% increased risk of lung cancer, dermal lesions, and bladder cancer, respectively) and Pb (BMDL_01_: 0.5 µg kg bw^−1^ day^−1^ for 1% increased risk of developmental neurotoxicity; BMDL_10_: 0.63 µg kg bw^−1^ day^−1^ for 10% increased risk of nephrotoxicity; BMDL_01_: 1.5 µg kg bw^−1^ day^−1^ for 1% increased risk of cardiovascular effects). Since total As from terrestrial livestock is estimated to be 70% in the inorganic form and BMDLs of As refer to *i*As (which is more toxic than the organic form), a percentage of 70% of the total As concentration measured in samples was presumed to be *i*As and, therefore, used for exposure calculation [31]. On the contrary, the total Hg concentrations measured in pig samples were assumed to be *i*Hg since the prevalence of the organic form in meat products derived from terrestrial livestock is negligible [32]. As for Cr, although Cr(VI) is much more toxic than Cr(III), it was assumed that the entire amount of Cr found in samples was trivalent since most of the ingested Cr(VI) is reduced to Cr(III) in the stomach and an HBGV has been established only for Cr(III) [33].

#### 2.5.2. Calculation of Dietary Intakes of TMMs

The daily (EDI) or weekly (EWI) intakes of the measured TMMs through fresh pig muscle (meat) or pig liver consumption were estimated by using a deterministic approach and by formulating two possible exposure scenarios: (i) a less-conservative scenario (hereafter referred to as middle-bound P50, MB-P50) by considering median chronic consumption data (P50) combined with the median middle-bound (MB) TMM contamination levels (where elemental concentrations below the MLODs were replaced by MLOD/2 values); (ii) a worst-case scenario (hereafter referred to as upper-bound P95, UB-P95), by considering 95th percentile (P95) chronic consumption data combined with the median upper-bound (UB) TMM contamination levels (where concentrations below the MLODs were replaced by MLOD values). In all cases, TMMs were assumed to be 100% bioaccessible, hence contamination levels were not converted and used as such.

The Italian population was selected as the target group since it is the major consumer of the analyzed pig meat and pig liver samples. Chronic consumption data were retrieved from the INRAN-SCAI-2005-06 Italian National Food Consumption Survey [34] which is included in the EFSA Comprehensive European Food Consumption Database [35]. Calculations of EDIs or EWIs were performed independently for three selected age classes: children (36 months–9 years), adolescents (10–17 years), and adults (18–64 years) [35].

#### 2.5.3. Health Risk Estimation

Once calculated, the EDIs or EWIs for Al, Cd, Cr, Cu, Fe, *i*Hg, Ni, Sn, U, and Zn were compared with their relative HBGVs to estimate the potential exposure to these food contaminants arising from only consumption of pig meat or livers. As recommended by EFSA, a margin of exposure (MOE) approach was instead adopted to assess the risk of *i*As and Pb, which have a non-threshold toxicological effect. MOEs were calculated by the ratio of BMDLs to the relative of EDIs and the results used as risk indexes to define levels of concern. In general, MOE values higher than 10,000 (if based on BMDLs from animal studies) indicate that the intake of the toxic substance is of low concern [28].

## 3. Results and Discussion

### 3.1. Contamination Levels of TMMs in Pig Muscle and Liver

With the aim to reduce the presence of TMMs in the feed and food chain to the lowest possible levels, monitoring programs pursuant to European legislation [36] are continuously carried out by all member states to ensure the compliance of food and feed with such chemical hazards as well as to identify and mitigate their sources of origin.

At the same time, maximum admissible levels have been set up in both feedstuffs and foodstuffs by the European Union [37,38]. Indeed, maximum levels of Pb and Cd and maximum residue levels of Cu and Hg (which are used as pesticides) have been established for pig meat and offal [38,39].

**Figure 1:**
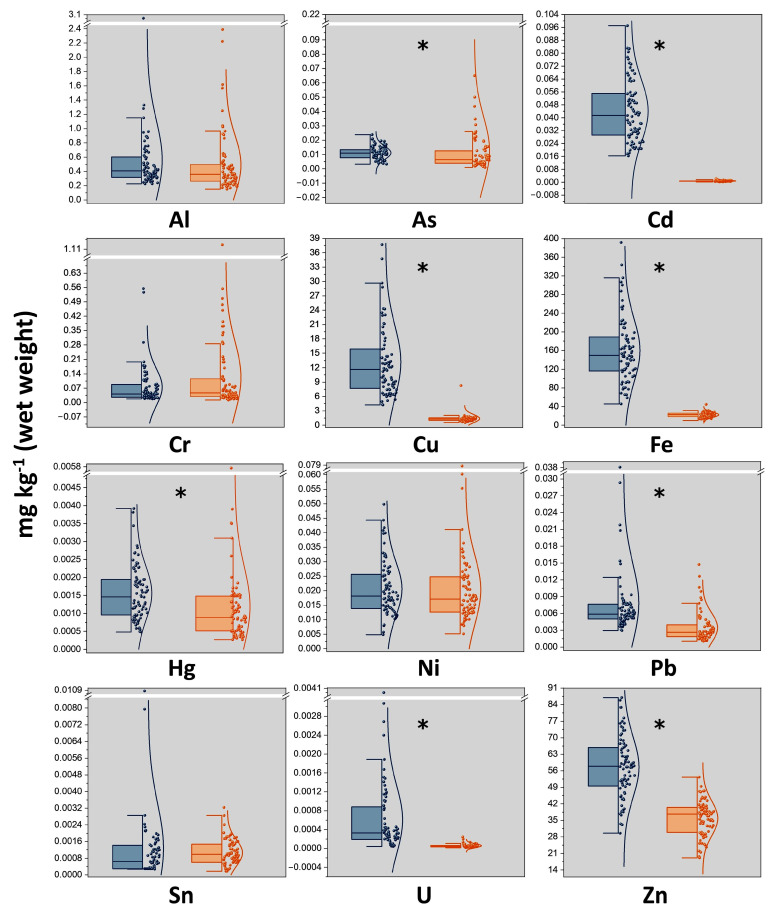
Half-box/violin plots showing concentrations (upper-bound, UB, wet weight) and size distributions of TMMs in pig liver (blue) and muscle (orange) samples. Boxes: lower 25% quartile, median, and upper 75% quartile; whiskers: ± 2 times the interquartile range; statistically significant differences between muscle and liver according to Mann–Whitney U test: * (*p* ≤ 0.01).

**Figure 2:**
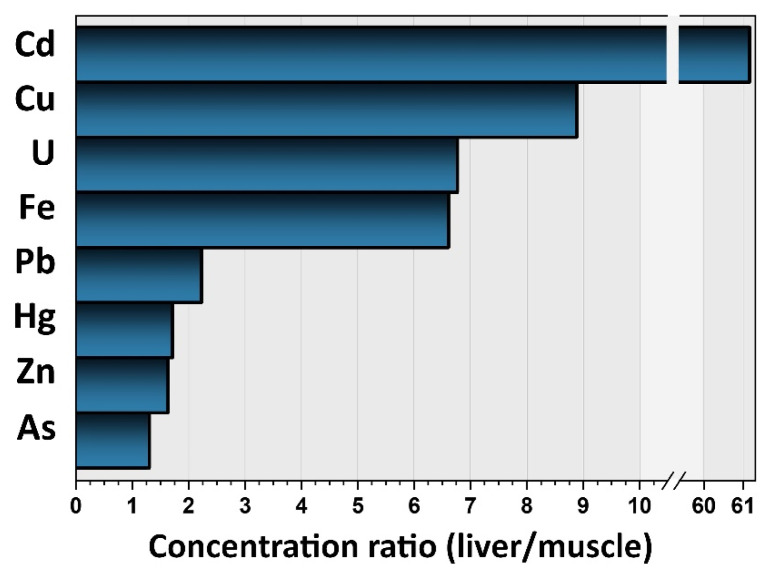
Concentration ratios (liver/muscles) of toxic metals and metalloids (TMMs) in the analyzed pig samples.

Occurrence data of TMMs in the pig muscles and pig livers analyzed in the present work are summarized in Figure 1 and detailed in Table S4 of the Supplementary Materials. The elements were found in the following order of abundance (median concentrations, mg kg^−1^, wet weight): Zn (38) > Fe (23) > Cu (1.3) > Al (0.36) > Cr (0.047) > Ni (0.017) > *i*As (0.0063) > *i*Hg (0.0033) > Pb (0.0026) > Sn (0.00099) > Cd (0.00068) > U (0.000048) in muscles; Fe (150) > Zn (58) > Cu (12) > Al (0.41) > Cr (0.042) > Cd (0.042) > Ni (0.018) > *i*As (0.011) > Pb (0.0059) > *i*Hg (0.0049) > Sn (0.00064) > U (0.00033) in livers. Except for Al, Cr, Ni, and Sn, all the measured TMM levels were significantly higher (*p* < 0.01) and more widely distributed in liver compared to muscle samples (Figure 1). This scattered distribution implies the existence of a high inter-animal variability among pigs which, besides deriving from feed, environment, and farming practices, may be linked to individual genetic factors [40,41]. As expected, Cu, Fe, and Zn were the prominent elements in both tissues, where they exert biologically important functions and are regulated by homeostatic processes [42]. In general, concentrations of these elements, together with those of Al, Cr, and Ni, were in line with those found in other countries such as China [11,43,44], Serbia [16,45], and Poland [46]. Comparison with literature data is, however, a complex task due to the contribution of many factors, among which is the type of muscle. For instance, Cu was reported to be less abundant in pectoral muscle than in diaphragm of cattle [47]. Wild boars and domestic pigs were reported to deposit two or three times the Cu in the liver compared to muscles [17,48], but, in the present investigation, a 9-fold higher concentration in the hepatic than in the muscle tissue was recorded (Figure 2). This, together with the fact that 3 out 80 (4%) liver samples exceed the maximum residue limits of Cu (as pesticide residue) of 30 mg kg^−1^ [39], may suggest a potential targeted accumulation in this organ, which, in turn, can be the consequence of its administration in intensive farming systems as mineral supplements.

Pb median concentrations were approximately 40 and 25 times below the maximum levels of 0.10 and 0.15 mg kg^−1^ established for muscle and liver samples, respectively [38]. Similarly, no muscle and liver samples exceeded the maximum levels of Cd of 0.050 and 0.50 mg kg^−1^ [38]. Interestingly, both Cd and Pb amounts were significantly lower than most found in previous studies. Nevertheless, it should be considered that animals analyzed by other authors were slaughtered at younger ages [13,14,16,18,43,49,50,51,52] and, hence, accumulated lower amounts of these contaminants. Cd showed a 60-fold higher median concentration in livers (Figure 2). In this context, it was demonstrated that feed supplements containing Fe, Zn, Cu, and Ca may significantly increase the rate of absorption and accumulation of Cd in livestock tissues, hence suggesting that the main source of Cd for pigs is feed itself [8,41]. Median concentrations of *i*As and *i*Hg in muscles (0.0064 and 0.00088 mg kg^−1^, respectively) were significantly lower compared to data reported by EFSA in livestock meat [31,32]. Specifically, *i*As was detected only in 76% of the muscle samples analyzed. This situation may reflect the decline in the use of both *i*As and *i*Hg compounds in agriculture practices over recent decades and the gradual phasing out of fish meal in pig diets which, together with drinking water, are reported as the main contamination sources in pig farming [10,53]. On the other hand, median concentrations of *i*As and *i*Hg found in livers (0.016 and 0.0015 mg kg^−1^, respectively) were higher than those listed by other authors [16,17]. This may support the hypothesis of a targeted accumulation in the hepatic tissue, potentially linked to the higher age and weight of the animals analyzed in the present study.

Eventually, the presence of U is also related to water contamination [54]. Nevertheless, U has been poorly investigated in meat and meat products, albeit meat, fish, and sausages were estimated to supply 8.5–12.4% of the overall dietary U intake [55]. Therefore, studies of U concentrations in pig meat and offal need to be deepened and further explored to clarify both natural and anthropogenic sources as well as its occurrence and co-occurrence with other TMMs in livestock food chains.

### 3.2. Evaluation of Human Dietary Exposure to TMMs

Intakes of TMMs from fresh pig meat and pig liver ingestion were calculated in order to investigate the impact exerted by consumption patterns of different Italian population age groups on the dietary exposure levels.

Median chronic consumption (P50) of pig meat by the Italian population (used for calculations) were of 1.19, 0.83, and 0.62 g kg bw^−1^ day ^−1^ for children, adolescents, and adults, while those of pig liver were 2.04, 1.04, and 0.78 g kg bw^−1^ day^−1^, respectively [35]. For the high consumers (P95), the chronic consumption data of pig meat were approximately twice as high as P50 consumption data for all the population groups, while those of pig liver were slightly higher only for adults, being 2.96, 2.04, and 1.44 g kg bw^−1^ day^−1^ of pig meat for children, adolescents, and adults, and 2.04, 1.04, and 0.97 g kg bw^−1^ day^−1^ of pig liver for children, adolescents, and adults, respectively [35].

The results of the multiplication of the above consumption data and the TMM concentration data experimentally measured in samples were reported as EDI or EWI of TMMs. The results are presented in Table 1 (alongside the reference toxicological values for each TMM for a rapid comparison).

As it can be noticed from Table 1, children’s EDIs and EWIs of all the TMMs were higher than for the adolescent group and, in turn, EDIs and EWIs for adolescents were higher than for the adult group, mostly because of the larger amount of foods eaten by the younger population in relation to the lower body weights.

The dietary exposure to Sn was the lowest one relative to the consumption of both meat and liver, with median intakes approximately 6–7 orders of magnitude lower than the reference PTWI of 14 mg kg bw^−1^ week^−1^. The low EWI is strongly driven by the low Sn content found in samples rather than food consumption rates, which reflects the low occurrence of this metal in livestock products. Indeed, human exposure to Sn is mainly attributable to ingestion of canned food, where Sn can migrate from the tin-lined cans into the food content [56]. Sn in the form of stannous (Sn II) chloride can also be used as a food additive (E 512) for stabilizing the color of some bottled or lacquered canned vegetables. Hence, dietary exposure to this compound has also been related to consumption of a few canned products, although no safety concern has been identified when used at authorized use levels [57]. On the other hand, the consumption of fish and seafood, especially when originating from highly contaminated areas, has been reported as the main route of exposure to organotin compounds [58]. These are released into the environment from anthropogenic sources and can accumulate in the aquatic chain at significant levels.

Intakes of Al, Cr, and U were very low. The maximum EWIs of Al were found in the UB-P95 exposure scenario for children (0.0058 mg kg bw^−1^ week^−1^) and adolescents (0.0030 mg kg bw^−1^ week^−1^) consuming pig liver, and for children (0.0030 mg kg bw^−1^ week^−1^) consuming pig meat according to the MB-P50 scenario (Table 1).

In general, most unprocessed foods do not contain more than 5 mg Al kg^−1^. Cereal, cereal products, beverages, and vegetables (rather than meat and other foods of animal origin) are known as the main contributors to the dietary exposure to Al of children and toddlers [59]. The EDI of U from pig livers was found to be slightly higher (1 order of magnitude) compared to the EDI from pig meat for all the population groups, both in the MB-P50 and UB-P95 scenario (Table 1). This is mainly due to the higher concentrations experimentally found in liver tissues, where it is likely to accumulate (Figure 1, Figure 2). Since referring to two single categories of foods, our results are significantly lower compared to the EDI of the European population to U through the whole diet, which was reported to range from 0.05 to a maximum of 0.14 g kg bw^−1^day^−1^ [54]. Even if the sum of the UB-P95 EDIs of U by children through pig meat (0.00014 µg kg bw^−1^ day^−1^) and pig liver (0.00067 µg kg bw^−1^ day^−1^) is taken into account, it would be equal to 0.00081 µg kg bw^−1^ day^−1^, which is 1000 times lower than the reference TDI of 0.6 µg kg bw^−1^ day^−1^. Actually, U in farmland soil is hardly mobilized from soil to crops, and it is even more so from crops to animals and derived foods [54].

Apparently, no worrying EDIs or EWIs of *i*As, *i*Hg, and Pb via meat or liver consumption were identified in all population age groups (Table 1). Indeed, cereal products were reported as the main contributors to the total dietary exposure to Pb [60]. Cereal products, together with foods for special dietary uses, bottled water, coffee, beer, rice grains, fish, fish products, and vegetable products, were also identified as main contributors to the exposure of the European population to *i*As [31]. For *i*Hg, fish and seafood are primarily responsible for most (approx. 25%) of the overall dietary exposure to this contaminant, while the contribution of meat and meat products is generally considered negligible [32].

Consumption of pig meat by children was associated with the highest exposures to Zn (0.045 mg kg bw^−1^ day^−1^ according to the MB-P50 scenario and 0.11 mg kg bw^−1^ day^−1^ according to the UB-P95 scenario) (Table 1). Interestingly, it can be also noticed that, whatever the exposure approach adopted, the highest ever exposure was to Cd by children through liver consumption (0.59 µg kg bw^−1^ week^−1^), in line with the median exposure levels reported in Europe [61]. In this case, edible organ meat (including pig livers) was identified among the food commodities contributing the most to the overall Cd dietary exposure [61].

In conclusion, estimates of dietary exposure to each TMM appear to be very low and far below the toxicological reference values or points when considering the only consumption of pig meat or pig liver. Nevertheless, since the hazard metrics (HBGVs and BMDLs) and the exposure metrics (EDI and EWI) are poorly informative and hardly interpretable when evaluated on their own, quantitative estimates of the potential harmful impact on human health of TMM intakes were finally performed by conducting a risk characterization.

### 3.3. Risk Characterization

For TMMs showing a toxic threshold effect, the ratios between the EDIs or EWIs and the relative HBGVs were used as metrics to quantify the risk arising from the specific consumption of pig meat and pig liver.

**Figure 3:**
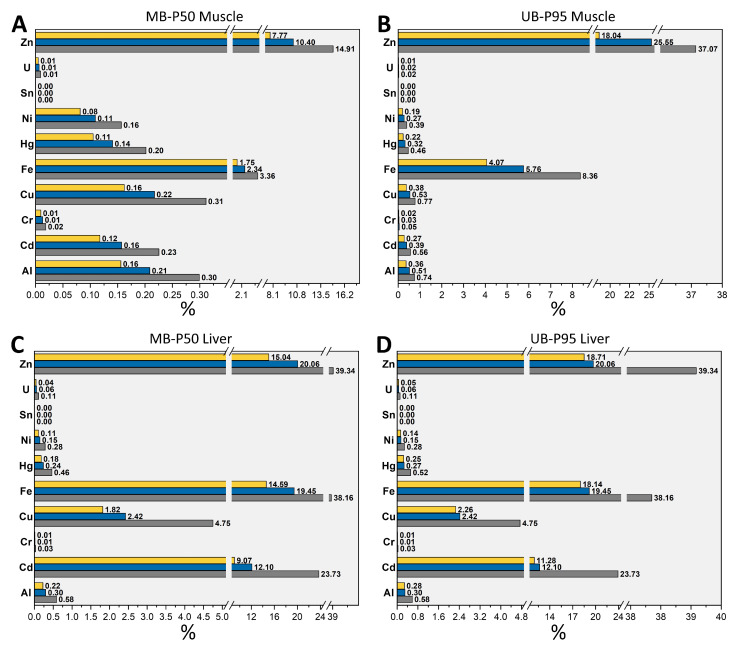
Contribution percentages of pig meat (**A**,**B**) and pig liver (**C**,**D**) consumption to the HBGVs of Al, Cd, Cr, Cu, Fe, *i*Hg, Ni, Sn, U, and Zn according to the estimated less pessimistic (MB-P50, **A**,**C**) and worst-case (UB-P95, **B**,**D**) dietary exposure scenarios for Italian population age groups (yellow bars: adults; blue bars: adolescents; gray bars: children).

Overall, the risk characterization analysis suggested that neither pig meat nor pig liver were responsible for the exceedance of the HGBVs, whatever the TMM and exposure scenario considered (Table 1). As can be observed from Figure 3, the health risk related to TMMs decreased with increasing population age, with children being the most vulnerable group. Indeed, contributions of both pig meat and pig liver consumption to all the HBGVs for the adult population were approximately just half as large as those for the child population. Of note, no differences in pig liver contributions between the MB-P50 (Figure 3C) and MB-P95 exposure scenarios (Figure 3D) were observed for children and adolescents, since for these two population groups pig liver consumption data at the two consumption levels were quantitatively the same (see Section 3.2). In detail, EDIs and EWIs of Al, Cr, Hg, Ni, Sn, and U for all the populations did not exceed 0.74% of the HBGVs even considering the UB-P95 worst-case exposure scenario. Therefore, the impact of meat and liver consumption was considered negligible and is not further discussed (Figure 3B,D).

By contrast, the EDIs (UB-P95) obtained for Fe represented a percentage of 4–8% (pig meat) and 18–38% (pig liver) of the PMTDI of 0.80 mg kg bw^−1^ day^−1^.

When considering the EDIs (UB-P95) of Zn by children, the maximum contributions of 37% and 39% of the PMTDI of 0.30 mg kg bw^−1^ day^−1^ by meat and liver, respectively, were observed (Figure 3B,D). If the simultaneous consumption of meat and liver by children is hence assumed, the individual contributions to PMDIs would add up and would result in alarming intake levels of Zn equivalent to 54% (MB-P50) and 75% (UB-P95) of the PMTDI.

With regard to the type of product considered, the EDIs and EWIs of Cu and Cd showed the overall highest variable contribution to the relative PMTDI or TWI, being significantly lower when considering muscle consumption and significantly higher when considering liver consumption (Figure 3A,B vs. Figure 3C,D). This result is due to the difference in the two metals in terms of concentrations between the muscular and hepatic tissues of pigs, the latter in which they tend to bioaccumulate (Figure 2). While there seems to be no particular concern about Cu intakes since all the population groups roughly absorb less than 50% of the overall Cu introduced with the diet [62], the same cannot be assumed for Cd intake, for which inconsistent data about its bioavailability to humans are still present. Cd exposure levels, especially during childhood, can be considered among the most alarming issues that emerged from this study. The 0.12–0.23% (MB-P50) and the 0.27–0.56 % (UB-P95) of the TWI of Cd (equal to 2.5 µg kg bw^−1^ week^−1^) are covered only by pig meat consumption, but these values rose drastically to 9–24% (MB-P50) and 11–24% (UB-P95) when the only consumption of pig fresh livers was considered. Meat and liver from Italian heavy pigs, besides being consumed as fresh unprocessed products, are widely destined for the dry curing industry for the preparation of several products (e.g., hams and dried cured products). The contribution to the Cd TWI of these products, as well as that of other food categories (i.e., cereals and cereal products, vegetables, nuts and pulses, and starchy roots), sum up together, suggesting that the Cd TWI could be exceeded though the whole diet. On the other side, considering the use of animal by-products as primary ingredients for the formulation of commercial pet foods, the same aspect may also pose a serious health threat for pets. Indeed, animal-based ingredients such as pig by-products were reported as contributing factors to pet food contamination by high concentrations of toxic metals [63,64].

**Figure 4:**
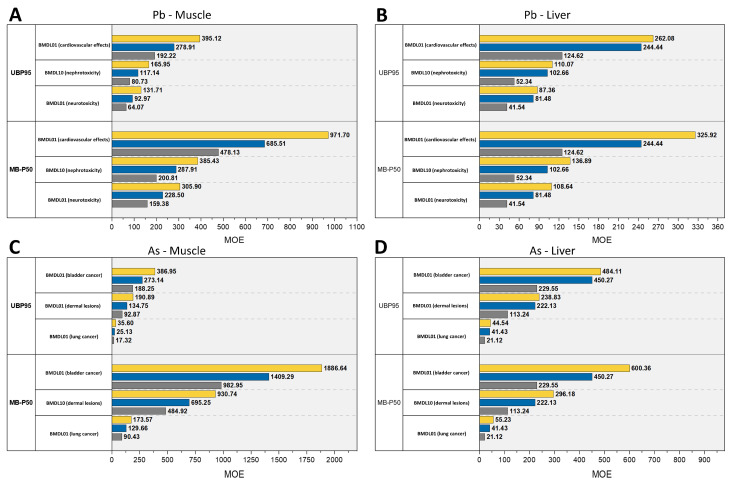
Comparison between margins of exposure (MOEs) to *i*As and Pb (dimensionless, calculated as the ratio between BMDLs and EDIs) for the Italian population age groups related to the consumption of pig meat (**A**,**B**) and pig liver (**C**,**D**) (yellow bars: adults; blue bars: adolescents; gray bars: children). MOEs were calculated according to the less pessimistic (MB-P50, **A**,**C**) and worst-case (UB-P95, **B**,**D**) dietary exposure scenarios.

With regard to *i*As and Pb, it was found that dietary intake estimates were all below the BMDLs (Table 1), whatever the exposure scenario and the population group considered. Therefore, the resultant ranges of MOEs were >1 (Figure 4), suggesting that exposure to these contaminants could be considered safe for children, adolescents, and adults. This is particularly true when the risk of cardiovascular effects and bladder cancer potentially induced by Pb and *i*As, respectively, via meat or liver ingestion is considered; indeed, the 1% increased risk of such adverse events (BMDLs_01_) was very low even in the UB-P95 exposure scenario, resulting in MOEs to Pb of 395–268 (adults), 278–244 (adolescents), and 192–125 (children) (muscle–liver, Figure 4A,B) and in MOEs to *i*As of 387–484 (adults), 273–450 (adolescents), 188–230 (children) (muscle–liver, Figure 4C,D).

Overall, significantly lower MOEs of both Pb and *i*As were observed for children compared to adults and from liver compared to muscle, except for the UB-P95 exposure to *i*As (Figure 4C vs. Figure 4D). In this case, the lower MOEs were mainly due to the higher dietary exposure values deriving from muscle consumption compared to liver (Table 1) which, in turn, were influenced by the significant number of missing values and their replacement with MLOD values (see Section 2.4 and Section 2.5.2). On the contrary, the lowest and the least safe MOEs to Pb and *i*As of children (calculated at the UB-P95 exposure levels) were related, respectively, to the 1% increased risk of neurotoxicity from pig liver consumption (MOE = 42, Figure 4B) and the 1% increased risk of lung cancer from pig meat consumption (MOE = 17, Figure 4C). Even though these dietary exposures of children to *i*As resulted in MOEs > 1, the whole diet and drinking water, when taken together as routes of exposure, could be supposed to further reduce MOEs to significantly lower levels [65]. In addition, albeit the period of exposure would be potentially limited to childhood lifespan, an increased risk of cancer in adulthood could still be associated with the exposure to the same *i*As during early stages of life [66]. The same considerations could be made for Pb: although dietary exposure of children to Pb resulted in MOEs > 1 and the risk of neurological development impairment could be considered low, the overall risk of adverse health effects could not be excluded due to the high vulnerability of children to toxic substances [60].

In closing, the consumption of fresh meat and liver from Italian heavy pigs may not be a serious health risk for local consumers as far as TMMs are concerned. Some threats, however, may arise from the dietary intake of Cd, As, Fe, and Zn, especially when pig liver consumption by children is considered, suggesting that, from a total diet point of view, the toxicological guidance values might be exceeded.

Notwithstanding the novelty of the results obtained, the present study is characterized by some constraints to be addressed by future research. First, a greater sample size and comparison with heavy pigs raised in countries other than Italy would be beneficial for confirming the finding achieved and gaining more insight on the influence of the overall environment and the farming practices on the accumulation of TMMs by livestock. At the same time, further elemental speciation studies are desirable to evaluate with greater accuracy the toxicological impact of certain TMMs. In fact, estimations of *i*As and *i*Hg dietary exposures carried out in this study are characterized by a significant margin of uncertainty: the literature-driven assumption that 70% of *i*As and 100% of *i*Hg concentrations were present in inorganic form may have led to biased (under- or over-) estimates of exposure levels to these contaminants. Lastly, a future evaluation of human exposure to TMMs by using probabilistic approaches is required in order to gain essential information about uncertainty and variability associated with exposure estimates, obtain more realistic results, and improve the overall risk assessment accuracy.

## 4. Conclusions

Contaminations levels of Al, As, Cd, Cr, Cu, Fe, Hg, Ni, Pb, Sn, U, and Zn in muscle and liver tissues of Italian heavy pigs were found to be very low, and, overall, quite in line with data reported by other countries in the world. A significant degree of accumulation of Cd and Cu in livers compared to muscle was observed. In this case, concentrations were found to be higher compared to literature data which, however, referred mainly to lean pigs. This suggested that heavier weights and higher age at the slaughter of heavy pigs could influence the accumulation of such elements in the hepatic tissue. The estimated intakes of all TMMs by children, adolescents, and adults from pig meat or pig liver consumption were also found to be very low. Nevertheless, from the risk characterization analysis, it emerged that high-consumer children may be exposed to Cd, Fe, and Zn amounts which, through the combined consumption of pig meat and pig livers, may contribute to more than 24, 46, and 76% of the tolerable weekly intakes of these elements. Since other foods from the total diet represent additional exposure sources of Cd, Fe, and Zn, it can be assumed that tolerable weekly intakes of these metals are widely exceeded though the whole diet and, therefore, some food safety concerns for the younger population arise.

In conclusion, the achieved results suggest the need to reduce as much as possible the carry-over and accumulation of TMMs from feedstuffs to animal tissues. In this context, integrated monitoring programs to check on the occurrence of these contaminants in soil, feeds, water, livestock, and related meat products should be stepped up, while the dietary intake needs should be more frequently evaluated. Within the risk management framework, the greater availability of information about these aspects would help regulators and decision makers to adopt both preventive and protective measures. Among these, action levels and target levels for TMMs could be established, in order to promptly identify and minimize sources of contamination. As regards risk communication, local consumers need to be educated to consume pig liver within reason. Hence, specific dietary consumption advice should be provided, especially to the most vulnerable categories of consumers such as children, the elderly, and pregnant and breastfeeding women.

## Figures and Tables

**Table 1:** Less pessimistic (MB-P50) ^a^ and worst-case (UB-P95) ^b^ estimated dietary exposure scenarios for Italian population to toxic metals and metalloids through pig meat and pig liver chronic consumption and relative HBGV/BMDL values established for each element.

			Exposure by Pig Meat			Exposure by Pig Liver		
TMM	HBGV/BMDL	Age Group		MB-P50 ^a^	UB-P95 ^b^		MB-P50 ^a^	UB-P95 ^b^
Al	TWI 1 mg kg bw^−1^ week^−1^	Children	EWI (mg kg bw^−1^ week^−1^)	2.99 × 10^−3^	7.44 × 10^−3^	EWI (mg kg bw^−1^ week^−1^)	5.83 × 10^−3^	5.83 × 10^−3^
		Adolescents		2.09 × 10^−3^	5.13 × 10^−3^		2.97 × 10^−3^	2.97 × 10^−3^
		Adults		1.56 × 10^−3^	3.62 × 10^−3^		2.23 × 10^−3^	2.77 × 10^−3^
*i*As	BDML01 0.69, 3.7, 7.5 µg kg bw^−1^ day^−1^	Children	EDI (µg kg bw^−1^ day^−1^)	7.63 × 10^−3^	3.98 × 10^−2^	EDI (µg kg bw^−1^ day^−1^)	3.27 × 10^−2^	3.27 × 10^−2^
		Adolescents		5.32 × 10^−3^	2.75 × 10^−2^		1.67 × 10^−2^	1.67 × 10^−2^
		Adults		3.98 × 10^−3^	1.94 × 10^−2^		1.25 × 10^−2^	1.55 × 10^−2^
Cd	TWI 2.5 µg kg bw^−1^ week^−1^	Children	EWI (µg kg bw^−1^ week^−1^)	5.64 × 10^−3^	1.40 × 10^−2^	EWI (µg kg bw^−1^ week^−1^)	5.93 × 10^−1^	5.93 × 10^−1^
		Adolescents		3.93 × 10^−3^	9.67 × 10^−3^		3.02 × 10^−1^	3.02 × 10^−1^
		Adults		2.94 × 10^−3^	6.82 × 10^−3^		2.27 × 10^−1^	2.82 × 10^−1^
Cr	TDI 0.3 mg kg bw^−1^ day^−1^	Children	EDI (mg kg bw^−1^ day^−1^)	5.62 × 10^−5^	1.40 × 10^−4^	EDI (mg kg bw^−1^ day^−1^)	8.48 × 10^−5^	8.48 × 10^−5^
		Adolescents		3.92 × 10^−5^	9.64 × 10^−4^		4.32 × 10^−5^	4.32 × 10^−5^
		Adults		2.93 × 10^−5^	6.81 × 10^−5^		3.24 × 10^−5^	4.03 × 10^−5^
Cu	PMTDI 0.5 mg kg bw^−1^ day^−1^	Children	EDI (mg kg bw^−1^ day^−1^)	1.56 × 10^−3^	3.87 × 10^−3^	EDI (mg kg bw^−1^ day^−1^)	2.38 × 10^−2^	2.38 × 10^−2^
		Adolescents		1.09 × 10^−3^	2.67 × 10^−3^		1.21 × 10^−2^	1.21 × 10^−2^
		Adults		8.11 × 10^−4^	1.88 × 10^−3^		9.08 × 10^−3^	1.13 × 10^−2^
Fe	PMTDI 0.8 mg kg bw^−1^ day^−1^	Children	EDI (mg kg bw^−1^ day^−1^)	2.69 × 10^−2^	6.69 × 10^−2^	EDI (mg kg bw^−1^ day^−1^)	3.05 × 10^−1^	3.05 × 10^−1^
		Adolescents		1.87 × 10^−2^	4.61 × 10^−2^		1.56 × 10^−1^	1.56 × 10^−1^
		Adults		1.40 × 10^−2^	3.25 × 10^−2^		1.17 × 10^−1^	1.45 × 10^−1^
*i*Hg	TWI 4 µg kg bw^−1^ week^−1^	Children	EWI (µg kg bw^−1^ week^−1^)	8.07 × 10^−3^	1.84 × 10^−2^	EWI (µg kg bw^−1^ week^−1^)	2.08 × 10^−2^	2.08 × 10^−2^
		Adolescents		5.63 × 10^−3^	1.27 × 10^−2^		9.48 × 10^−3^	1.06 × 10^−2^
		Adults		4.21 × 10^−3^	8.95 × 10^−3^		7.11 × 10^−3^	9.90 × 10^−3^
Ni	TDI 13 µg kg bw^−1^ day^−1^	Children	EDI (µg kg bw^−1^ day^−1^)	2.04 × 10^−2^	5.70 × 10^−2^	EDI (µg kg bw^−1^ day^−1^)	3.70 × 10^−2^	3.70 × 10^−2^
		Adolescents		1.42 × 10^−2^	3.49 × 10^−2^		1.89 × 10^−2^	1.89 × 10^−2^
		Adults		1.06 × 10^−2^	2.47 × 10^−2^		1.42 × 10^−2^	1.76 × 10^−2^
Pb	BMDL01/10/01 0.5/0.63/1.5 µg kg bw^−1^ day^−1^	Children	EDI (µg kg bw^−1^ day^−1^)	3.14 × 10^−3^	7.80 × 10^−3^	EDI (µg kg bw^−1^ day^−1^)	1.20 × 10^−2^	1.20 × 10^−2^
		Adolescents		2.19 × 10^−3^	5.38 × 10^−3^		6.14 × 10^−3^	6.14 × 10^−3^
		Adults		1.63 × 10^−3^	3.80 × 10^−3^		4.60 × 10^−3^	5.72 × 10^−3^
U	TDI 0.6 µg kg bw^−1^ day^−1^	Children	EDI (µg kg bw^−1^ day^−1^)	5.76 × 10^−5^	1.43 × 10^−4^	EDI (µg kg bw^−1^ day^−1^)	6.70 × 10^−4^	6.70 × 10^−4^
		Adolescents		4.02 × 10^−5^	9.87 × 10^−5^		3.41 × 10^−4^	3.41 × 10^−4^
		Adults		3.00 × 10^−5^	6.97 × 10^−5^		2.56 × 10^−4^	3.18 × 10^−4^
Zn	PMTDI 0.3 mg kg bw^−1^ day^−1^	Children	EDI (mg kg bw^−1^ day^−1^)	4.47 × 10^−2^	1.11 × 10^−1^	EDI (mg kg bw^−1^ day^−1^)	1.18 × 10^−1^	1.18 × 10^−1^
		Adolescents		3.12 × 10^−2^	7.67 × 10^−2^		6.02 × 10^−2^	6.02 × 10^−2^
		Adults		2.33 × 10^−2^	5.41 × 10^−2^		4.51 × 10^−2^	5.61 × 10^−2^

HBGV/BMDL: health-based guidance value or lower limit of the benchmark dose; TWI: tolerable weekly intake; TDI: tolerable daily intake; PMTDI: provisional maximum tolerable daily intake; PTWI: provisional tolerable weekly intake; EWI: estimated weekly intake; EDI: estimated daily intake. ^a^ MB-P50: middle-bound concentration values combined with median (P50) chronic consumption data, as reported in Section 2.5.2. ^b^ UB-P95: upper-bound concentration values combined with P95 chronic consumption data, as reported in Section 2.5.2.

## Data Availability

The data presented in this study are available on request from the corresponding author.

## Supplementary Materials

The following supporting information can be downloaded at: https://www.mdpi.com/article/10.3390/foods11162530/s1, Table S1: Agilent 7900 ICP-MS operating conditions; Table S2: Mean concentration determinations, % recovery, and measurement precision (% RSD) for investigated analytes in certified reference materials (CRMs); Table S3: Analyte and isotopes, cell mode, normalized calibration slopes (NCS, L µg^−1^), and detection limits of the method (MLODs, µg kg^−1^) with the use of Rh as internal standard; Table S4: Upper bound concentrations (mg kg^−1^ wet weight) of TMMs in muscle and liver tissue of 80 Italian heavy pigs obtained by ICP-MS and mercury analyzer (*i*Hg).
