# Supplementary material for: Occurrence of Toxic Metals and Metalloids in Muscle and Liver of Italian Heavy Pigs and Potential Health Risk Associated with Dietary Exposure
Source: Foods. 2022 Aug 21;11(16):2530. doi: 10.3390/foods11162530 (PMC9407101; doi:10.3390/foods11162530)
Supplement: Supplementary file 1 [file foods-11-02530-s001.zip › foods-1849095-supplementary.pdf]

SUPPLEMENTARY MATERIAL

Article

Occurrence of Toxic Metals and Metalloids in Muscle and Liver of Italian Heavy Pigs and Potential Health Risk Associated with Dietary Exposure

Sergio Ghidini [1], Maria Olga Varrà [1,*], Lenka Husáková [2], Giovanni Loris Alborali [3], Jan Patočka [2], Adriana Ianieri [1] and Emanuela Zanardi [1]

[1] Department of Food and Drug, University of Parma, Strada del Taglio 10, 43126 Parma, Italy

[2] Department of Analytical Chemistry, Faculty of Chemical Technology, University of Pardubice, Studentska 573 HB/D, Pardubice, CZ-532 10, Czech Republic

[3] Headquarters, Istituto Zooprofilattico Sperimentale della Lombardia e dell'Emilia-Romagna, Via A. Bianchi 9, 25124 Brescia, Italy

* Correspondence: mariaolga.varra@unipr.it; Tel.: +39-0521-902-753

Table S1.

Agilent 7900 ICP-MS operating conditions.

Parameter	Setting		
ICP			
Plasma mode	General purpose		
Rf power (27 MHz) (W)	1550		
Sampling depth (mm)	10		
Plasma gas flow (L min^{-1})	15		
Auxiliary gas flow (L min^{-1})	0.9		
Nebulizer gas flow (L min^{-1})	1.05		
Nebulizer pump (rps)	0.1		
Spray chamber temperature (°C)	2		
Mass spectrometer	No gas mode	He mode	HEHe mode [a]
Extract 1 (V)		0	
Extract 2 (V)	-250	-245	-250
Omega bias (V)	-100	-120	-110
Omega lens (V)	9.7	12.7	12.3
Cell entrance	-30	-40	-140
Cell exit	-50	-60	-150
Deflect (V)	11.6	1.6	-60
Plate bias	-35	-60	-150
Helium flow (mL min^{-1})	0	6	10
OctP bias	-8	-18	-100
OctP RF		200	
Energy discrimination (V)	5	5	9
Number of elements	38 [b]	12 [c]	4 [d]
Acquisition			
Points per peak	1		
Replicates	3		
Sweeps/replicate	100		
Total acquisition time (s)	75		

[a] HEHe mode - high energy helium mode; Monitored isotopes (integration time): [b] ^{7}Li, ^{11}B, ^{24}Mg, ^{66}Zn, ^{85}Rb, ^{88}Sr, ^{89}Y, ^{90}Zr, ^{95}Mo, ^{101}Ru, ^{103}Rh, ^{105}Pd, ^{111}Cd, ^{118}Sn, ^{121}Sb, ^{133}Cs, ^{138}Ba, ^{139}La, ^{140}Ce, ^{141}Pr, ^{146}Nd, ^{147}Sm, ^{153}Eu, ^{157}Gd, ^{159}Tb, ^{163}Dy,^{165}Ho, ^{166}Er, ^{172}Yb, ^{175}Lu, ^{178}Hf, ^{185}Re, ^{195}Pt, ^{205}Tl, $^{206+207+208}$Pb, ^{209}Bi, ^{232}Th, ^{238}U (all 0.1 s); [c] ^{23}Na (0.3 s), ^{27}Al (0.1 s), ^{39}K, ^{44}Ca (both 0.3 s), ^{51}V (1 s), ^{52}Cr, ^{55}Mn, ^{56}Fe, ^{59}Co, ^{60}Ni, ^{63}Cu, ^{103}Rh (all 0.3 s); [d] ^{31}P (0.1 s), ^{75}As, ^{78}Se (both 1 s), ^{103}Rh (0.3 s).

Table S2.

Mean concentration determinations, % recovery and measurement precision (% RSD) for investigated analytes in certified reference materials (CRMs).

Element	CRM	Certified value (mg kg^{-1})	Measured value[a] (mg kg^{-1})	Recovery[b] (%)	RSD (%)	
					Intraday	Interday
Al	CRM 12-2-04 Wheat bread flour	3	3.2 ± 0.2	106	2.67	4.62
	CRM12-2-03 Lucerne	330	365 ± 6	111	0.85	8.35
As	BCR-CRM 185 Bovine Liver	0.024 ± 0.003	0.0242 ± 0.003	101	6.81	2.69
	NIST 1566 Oyster Tissue	13.4 ± 1.9	12.5 ± 1.2	93	4.74	2.07
	CRM 12-2-01 Bovine Liver	0.110 ± 0.016	0.116 ± 0.008	105	3.48	2.32
	CRM 12-2-04 Wheat bread flour	0.017 ± 0.0046	0.0184 ± 0.003	108	8.12	9.52
	CRM12-2-03 Lucerne	0.262 ± 0.020	0.292 ± 0.009	111	1.58	1.31
	NCS ZC 73015 Milk Powder	31 ± 7	29.7 ± 0.2	96	0.30	5.64
Cd	BCR 184 Bovine muscle	0.013 ± 0.002	0.0131 ± 0.003	101	9.68	6.07
	BCR-CRM 185 Bovine Liver	0.298 25	0.296 ± 0.042	99	7.12	2.28
	NIST 1577 Bovine Liver	0.097 14	0.098 ± 0.004	101	2.05	4.09
	NIST 1566 Oyster Tissue	3.5 ± 0.4	3.24 ± 0.03	93	0.44	2.12
	CRM 12-2-01 Bovine Liver	0.48 ± 0.03	0.46 ± 0.02	95	1.89	2.50
	CRM 12-2-04 Wheat bread flour	0.0415 32	0.0381 ± 0.004	92	4.99	0.53
	CRM12-2-03 Lucerne	0.136 ± 0.0065	0.124 ± 0.005	91	1.84	1.70
Cr	BCR 184 Bovine muscle	0.076	0.074 ± 0.002	99	1.28	5.85
	NIST 1577 Bovine Liver	0.053 ± 0.014	0.053 ± 0.009	100	8.86	3.75
	CRM 12-2-01 Bovine Liver	0.044	0.044 ± 0.01	100	12.1	7.86
	NCS ZC 73015 Milk Powder	0.39 ± 0.04	0.38 ± 0.02	97	2.37	4.87
	CRM12-2-03 Lucerne	0.900	0.89 ± 0.05	99	2.75	4.90
Cu	BCR 184 Bovine muscle	2.36 ± 0.06[c]	2.14 ± 0.16	91	3.79	1.99
	BCR-CRM 185R Bovine Liver	189 ± 4	180 ± 6	95	1.69	4.23
	NIST 1577 Bovine Liver	275.2 ± 4.6	253 ± 9	92	1.78	1.06
	NIST 1566 Oyster Tissue	63.0 ± 3.5	55.7 ± 0.4	89	0.32	0.42
	CRM 12-2-01 Bovine Liver	26.3 ± 1.6	25.1 ± 0.49	98	0.98	2.96
	NCS ZC 73015 Milk Powder	0.51 ± 0.13	0.48 ± 0.02	94	1.88	5.36
	CRM 12-2-04 Wheat bread flour	2.77 ± 0.03	2.69 ± 0.30	97	5.64	2.87
	CRM12-2-03 Lucerne	11.7 ± 0.75	11.4 ± 1.4	97	6.14	3.19
Fe	BCR 184 Bovine muscle	79 ± 2	73.1 ± 8.9	93	6.11	2.56
	BCR-CRM 185R Bovine Liver	214 ± 5	197 ± 23	92	5.85	2.25
	NIST 1577 Bovine Liver	197.94 ± 0.65	189 ± 10	96	2.75	1.23
	NIST 1566 Oyster Tissue	195 ± 34	189 ± 26	97	6.88	2.13
	CRM 12-2-01 Bovine Liver	495 ± 28	491 ± 59	99	6.01	5.23
	NCS ZC 73015 Milk Powder	7.8 ± 1.3	6.9 ± 0.5	89	3.48	6.12
	CRM 12-2-04 Wheat bread flour	23.8 ± 1.5	24.4 ± 1.9	102	3.99	1.81
	CRM12-2-03 Lucerne	355 ± 18	371 ± 21	104	2.77	1.42

Table S2. Continued

Element	CRM	Certified value (mg kg^{-1})	Measured value[a] (mg kg^{-1})	Recovery[b] (%)	RSD (%)	
					Intraday	Interday
Hg	BCR 185 Bovine Liver	0.044 ± 0.003	0.0460 ± 0.0005	105	0.54	[d]
	NIST 1577c Bovine Liver	5.36 ± 0.17[c]	5.0 ± 0.4[c]	93	3.80	7.06
	NIST 1566 Oyster Tissue	0.057 ± 0.015	0.0528 ± 0.0004	93	0.38	[d]
	CRM 12-2-01 Bovine Liver	0.37 ± 0.02	0.35 ± 0.02	95	3.14	5.92
Ni	BCR 184 Bovine muscle	0.270	0.265 ± 0.017	98	3.21	7.83
	NIST 1566 Oyster Tissue	1.03 ± 0.19	0.99 ± 0.04	96	1.88	3.42
	CRM 12-2-04 Wheat bread flour	0.3	0.28 ± 0.03	93	4.64	7.52
	CRM12-2-03 Lucerne	2.54 ± 0.18	2.81 ± 0.17	111	3.03	5.65
Pb	CRM 12-2-04 Wheat bread flour	0.041 ± 0.0078	0.038 ± 0.002	93	2.62	7.23
	NIST 1577 Bovine Liver	0.0628 ± 0.002	0.068 ± 0.002	109	1.14	1.62
	BCR 184 Bovine muscle	0.239 ± 0.011	0.250 ± 0.026	104	5.28	5.53
	BCR-CRM 185 Bovine Liver	0.501 ± 0.027	0.510 ± 0.048[c]	102	4.71	7.23
	NIST 1566 Oyster Tissue	0.480 ± 0.040	0.462 ± 0.010	96	1.07	2.52
	CRM 12-2-01 Bovine Liver	0.71 ± 0.08	0.71 ± 0.11	100	7.56	2.93
	NCS ZC 73015 Milk Powder	0.07 ± 0.02	0.069 ± 0.004	99	2.68	8.35
	CRM12-2-03 Lucerne	1.84 ± 0.17	2.0 ± 0.1	109	2.48	4.75
Sn	CRM 12-2-04 Wheat bread flour	< 3	0.30 ± 0.03	[d]	4.67	7.22
U	NIST 1566 Oyster Tissue	0.116 ± 0.006	0.115 ± 0.007	99	3.18	4.16
	NCS ZC 73015 Milk Powder	3[c]	3.05 ± 0.12[c]	102	1.95	4.38
Zn	BCR 184 Bovine muscle	166 ± 3	149.3 ± 0.8	90	0.25	1.32
	BCR-CRM 185 Bovine Liver	142 ± 3	132 ± 4	93	1.37	3.63
	NIST 1577 Bovine Liver	181.1 ± 1.0	168 ± 4	93	1.00	1.38
	NIST 1566 Oyster Tissue	852 ± 14	766 ± 17	90	1.11	0.73
	CRM 12-2-01 Bovine Liver	162 ± 6	152 ± 8	94	2.58	3.45
	NCS ZC 73015 Milk Powder	34 ± 2	33.3 ± 1.2	98	1.76	1.57

[a] Mean ± 2 S.D. (n = 3).
[b] Recovery (%) = (Found value/Declared value)×100.
[c] μg/kg
[d] Not determined.

Table S3.

Analyte and isotopes, cell mode, normalized calibration slopes (NCS, L μg^{-1}), and detection limits of the method (MLODs, μg kg^{-1}) with the use of Rh as internal standard.

Element	Isotope	Cell mode	NCS	MLOD[c]
Al	27	He	2.5×10^{-5}	349
As	75	HE He	5.2×10^{-4}	15
Cd	111	No gas	5.4×10^{-3}	0.24
Cr	52	He	6.7×10^{-3}	0.55
Cu	63	He	1.6×10^{-2}	6.8
Fe	56	He	4.5×10^{-3}	15
Hg [a]	–	–	2.8×10^{-2}	0.2
Ni	60	He	4.9×10^{-3}	4.4
Pb [b]	b	No gas	3.8×10^{-2}	0.70
Sn	118	No gas	1.5×10^{-2}	1.0
U	238	No gas	3.1×10^{-2}	0.074
Zn	66	No gas	5.9×10^{-3}	68

[a] Values were evaluated for direct analysis of Hg by single purpose atomic absorption spectrometer AMA 254.

[b] Pb is measured as the sum of the three most abundant isotopes, $^{206}Pb^{+}$, $^{207}Pb^{+}$ and $^{208}Pb^{+}$.

[c] Detection limits are corrected for initial sample weight and final volume and reflect the actual concentration in the undigested freeze-dried samples.

Table S4.

Upper Bound concentrations (mg kg^{-1} wet weight) of TMMs in muscle and liver tissue of 80 Italian heavy pigs obtained by ICP-MS and Mercury Analyzer (*i*Hg).

	Muscles					Livers				
Element	Min[a]	Q1[b]	Median	Q3[b]	Max[a]	Min[a]	Q1[b]	Median	Q3[b]	Max[a]
Al	0.15	0.26	0.36	0.50	2.4	0.23	0.32	0.41	0.60	4.3
*i*As	0.0010	0.0040	0.0063	0.013	0.20	0.0033	0.0077	0.011	0.013	0.024
Cd	0.00027	0.00053	0.00068	0.00090	0.0022	0.016	0.029	0.042	0.055	0.097
Cr	0.012	0.029	0.047	0.12	1.1	0.018	0.026	0.042	0.089	0.55
Cu	0.53	1.03	1.3	1.5	8.3	4.2	7.6	12	16	41
Fe	10	18	23	26	45	46	115	150	191	392
*i*Hg	0.0012	0.0019	0.0033	0.0055	0.027	0.0016	0.0032	0.0049	0.0065	0.013
Ni	0.0051	0.013	0.017	0.025	0.079	0.0048	0.014	0.018	0.026	0.050
Pb	0.0011	0.0019	0.0026	0.0040	0.015	0.0030	0.0050	0.0059	0.0077	0.048
Sn	0.00018	0.00060	0.00099	0.0015	0.0032	0.00028	0.00030	0.00064	0.0014	0.015
U	0.000017	0.000037	0.000048	0.000065	0.00025	0.000043	0.00019	0.00033	0.00090	0.0041
Zn	19	30	38	40	53	30	49	58	66	87

[a] Min–Max: minimum and maximum values found.

[b]Q1–Q3: first (25th percentile) and third (75th percentile) quartiles.
